# On Calculating Free Energy Differences Using Ensembles of Transition Paths

**DOI:** 10.3389/fmolb.2020.00106

**Published:** 2020-06-05

**Authors:** Robert Hall, Tom Dixon, Alex Dickson

**Affiliations:** ^1^Department of Biochemistry & Molecular Biology, Michigan State University, East Lansing, MI, United States; ^2^Department of Computational Mathematics, Science and Engineering, Michigan State University, East Lansing, MI, United States

**Keywords:** free energy, molecular dynamics, enhanced sampling, binding kinetics, statistical mechanics, nonequilibrium

## Abstract

The free energy of a process is the fundamental quantity that determines its spontaneity or propensity at a given temperature. In particular, the binding free energy of a drug candidate to its biomolecular target is used as an objective quantity in drug design. Recently, binding kinetics—rates of association (*k*_on_) and dissociation (*k*_off_)—have also demonstrated utility for their ability to predict efficacy and in some cases have been shown to be more predictive than the binding free energy alone. Some methods exist to calculate binding kinetics from molecular simulations, although these are typically more difficult to calculate than the binding affinity as they depend on details of the transition path ensemble. Assessing these rate constants can be difficult, due to uncertainty in the definition of the bound and unbound states, large error bars and the lack of experimental data. As an additional consistency check, rate constants from simulation can be used to calculate free energies (using the log of their ratio) which can then be compared to free energies obtained experimentally or using alchemical free energy perturbation. However, in this calculation it is not straightforward to account for common, practical details such as the finite simulation volume or the particular definition of the “bound” and “unbound” states. Here we derive a set of correction terms that can be applied to calculations of binding free energies using full reactive trajectories. We apply these correction terms to revisit the calculation of binding free energies from rate constants for a host-guest system that was part of a blind prediction challenge, where significant deviations were observed between free energies calculated with rate ratios and those calculated from alchemical perturbation. The correction terms combine to significantly decrease the error with respect to computational benchmarks, from 3.4 to 0.76 kcal/mol. Although these terms were derived with weighted ensemble simulations in mind, some of the correction terms are generally applicable to free energies calculated using physical pathways via methods such as Markov state modeling, metadynamics, milestoning, or umbrella sampling.

## 1. Introduction

In recent years there is a growing appreciation for the utility of binding kinetics in the prediction of drug efficacy (Lu and Tonge, 2010; Carroll et al., 2012; Vauquelin et al., 2012; Pei et al., 2014; Ayaz et al., 2016; Copeland, 2016; Costa et al., 2016; Guo et al., 2016; Tonge, 2017; Bruce et al., 2018; Lee et al., 2019; Nunes-Alves et al., 2020). Pharmacokinetic and pharmacodynamic models of drug activity in the body are inherently out of equilibrium: a drug is administered, it is absorbed, distributed to different tissues, metabolized and eliminated from the body. As such, kinetic constants of binding and release—beyond just the equilibrium constants of binding—are required to model drug action when the timescales of binding and release cannot be separated from the other competing processes (Bernetti et al., 2017). The relationship between molecular structure and the kinetics of binding (also called “structure-kinetic relationships” or SKR) is complicated, as small changes to structure can change kinetic constants by orders of magnitude (Ayaz et al., 2016). It is important to note that changes in kinetics are not always tied to changes in affinity (Guo et al., 2014), and that to accurately predict changes in kinetics, models of the ligand-binding transition state are needed to estimate transition-state stabilization or destabilization (Spagnuolo et al., 2017).

Computational methods that reveal structures of transition states and calculate binding (*k*_on_) and unbinding (*k*_off_) rate constants for real compounds are in their infancy, but are quickly developing (Dickson et al., 2017). It is a tremendous challenge to obtain reliable values for these quantities, as (1) they depend on the entire (un)binding pathway, not just its endpoints, and (2) the timescales of ligand binding and release often exceed the capabilities of molecular dynamics simulations by orders of magnitude. Specialized computing platforms have been applied to generate continuous binding pathways (Dror et al., 2011), although the unbinding process is typically beyond the reach of molecular dynamics simulation for compounds beyond millimolar drug fragments (Guo et al., 2016; Pan et al., 2017). Recent studies have used enhanced sampling methods in molecular dynamics to simulate ligand (un)binding pathways and determine mechanisms and rate constants *k*_on_ and *k*_off_ (Casasnovas et al., 2017; Tiwary et al., 2017; Dickson, 2018; Kokh et al., 2018; Lotz and Dickson, 2018; Bruno et al., 2019; Deb and Frank, 2019; Kirberger et al., 2019; Dixon et al., 2020). Some of these rate constants have shown surprisingly good agreement with experiment—given the extraordinarily long timescales involved—however these have the confounding uncertainty of force field accuracy (Yin et al., 2017; Camilloni and Pietrucci, 2018), there is a possibility for fortuitous cancelation of error. Unfortunately, the computational cost required to predict these quantities is typically massive (Camilloni and Pietrucci, 2018), especially for large protein systems and ligands with extremely long residence times, precluding the study of these events under a series of different simulation conditions (e.g., forcefields, water models, polarizability).

In the field of biomolecular modeling, blind challenges—where a series of objectives are released by the organizers, and participants entries are directly judged by their agreement with experiment—have been useful catalysts for the development of predictive algorithms (Lensink et al., 2017; Synapse, 2018; Croll et al., 2019; Parks et al., 2020). Although no blind challenge currently exists for the prediction of *k*_on_ and *k*_off_, we recently participated in the SAMPL6 SAMPLing challenge, which required participants to compute free energies as a function of simulation time and to compare the computational cost of different free energy calculation methods (Dixon et al., 2018; Rizzi et al., 2018, 2020). This challenge allows sampling methods to be assessed independently of force field accuracy, as all entries used the same initial coordinates, force field parameters and partial charges. Importantly, the challenge makes use of very small model systems (host-guest) that require considerably less computational resources to simulate, which allowed us to efficiently simulate binding and release for a number of systems, determine *k*_on_ and *k*_off_, and predict values for the binding free energy (Δ*G*) that would then be compared to experimental observables, as well as results from alchemical free energy perturbation methods (Gilson et al., 1997; Shirts and Chodera, 2008).

The standard free energy of binding was determined as a function of rate constants:

(1) $$\begin{array}{l} \Delta G=-k_{B}T\ln\;\frac{C^{0}k_{\text{on}}}{k_{\text{off}}} \end{array}$$

where *C*^0^ is a reference concentration of 1 mol/L. In this paper, we revisit this equation in detail and explicitly examine the assumptions made when the rate constants used in Equation (1) are computed through typical simulations with finite box-size and periodic boundary conditions. In section 3.1, we derive three correction terms that can be easily computed and facilitate a better connection with both experiment and alchemical computational free energy calculations. One term accounts for the particular definitions of the bound and unbound states. The second term accounts for residual electrostatic interactions that might still be present between the molecules, which is especially useful if one or both of the molecules carry an explicit charge. The third term accounts for the volume of the unbound state in the simulation box, which is useful to keep the simulated volume as small as possible during rate calculations. These terms were derived particularly with weighted ensemble simulations in mind, where rates are computed using the trajectory flux between two non-equilibrium ensembles. However, the second and third term can be directly applied to other simulation methods which employ physical simulation of the transition path ensemble, such as Markov state modeling (Singhal et al., 2004; Gu et al., 2014), metadynamics (Laio and Parrinello, 2002; Tiwary et al., 2017), milestoning (Faradjian and Elber, 2004; Votapka et al., 2017), and umbrella sampling (Torrie and Valleau, 1977; Nishikawa et al., 2018).

To examine questions of convergence, we reproduce our binding and unbinding simulations for a host-guest system with larger numbers of replicas and longer simulation times. We also explore the effects of the Langevin integrator on the prediction of unbinding and binding rates; in particular, how altering the friction coefficient (γ), defined in the Langevin integrator, impacts the binding and release processes. Although γ does not appear in the internal energy function, and hence cannot affect thermodynamic properties such as the binding free energy, we examine whether lower friction coefficients can accelerate the convergence of unbinding simulations.

## 2. Methods

### 2.1. Host-Guest Systems

**Figure 1:**
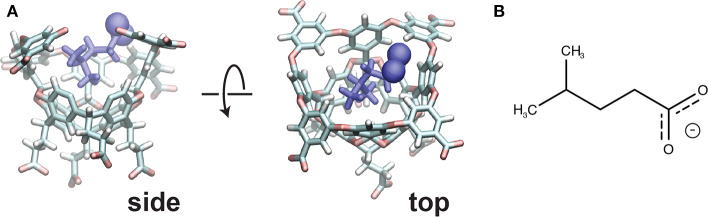
**(A)** The initial pose for the OA-G6 system (side view: left, top view: right). Note that some atoms from the host are removed in the side view for clarity. The carboxyl oxygens are shown in sphere representation. **(B)** The chemical structure of the G6 ligand in the deprotonated form.

The host-guest system utilized in this study is referred to as “OA-G6” (Figure 1), where the host is a Gibb deep cavity cavitand, referred to as an “octa acid” or “OA” (Gan et al., 2011). OA forms a basket-like structure with 4-fold symmetry, functionalized with four benzoic-acid substituents on the top rim of the basket and four more on the bottom. The guest ligand we study here is 4-methyl pentanoic acid (referred to as “G6”). This ligand harbors a negative charge at the carboxyl end of the alkyl chain.

### 2.2. Molecular Dynamics

The OA-G6 configuration was obtained from the organizers of the SAMPLing challenge (Rizzi et al., 2020). The system was solvated in a (roughly) cubic box with box length 4.28, 4.33, and 4.33 nm in the *x*, *y* and *z* dimensions, respectively. The system provided had a total of 7,976 atoms: 2,586 water molecules to solvate the system, 12 sodium and 3 chloride ions to neutralize the system, and the remaining atoms belonging to either the host or the guest. Forcefield parameters for the system are as provided by the original organizers of the SAMPLing challenge (Rizzi et al., 2018). The system was parameterized using GAFF (Wang et al., 2004) and then converted into Gromacs format. The conversion was done using ParmEd version 2.7.3. OpenMM v7.2.1 (Eastman et al., 2017) was used to run dynamics with the CUDA v9.0.176 platform. A Monte Carlo barostat is used to maintain a constant pressure of 1 atm. A timestep of 2 fs was used across all simulations.

We utilize the Langevin integrator, which uses a drag term and a noise term to account for the friction of solvent molecules and high velocity collisions that perturb the system. Langevin dynamics allows for the temperature to be controlled and can be used as a thermostat; we run all dynamics here at 300 K. Our host-guest system is propagated with the Langevin equation, shown below:

(2) $$\begin{array}{l} F=ma=-\nabla U\left(r\right)-m\gamma v+\sqrt{\frac{2m\gamma k_{B}T}{\tau }}R\left(t\right) \end{array}$$

where *U*(***r***) is the particle interaction potential, ***R***(*t*) is a random Gaussian noise term evaluated every timestep, *T* is the temperature, *k*_*B*_ is the Boltzmann constant, τ is the timestep and γ is the friction coefficient in units of inverse time. The friction term plays two different roles here, both modulating the second “drag” term, and the Gaussian noise. As γ approaches zero, the noise gets weaker and the dynamics becomes more deterministic. Here we run binding and unbinding simulations with γ values of 1.0, 0.1, and 0.01 ps^−1^.

### 2.3. Reweighting of Ensembles by Variance Optimization

To generate an ensemble of ligand unbinding events, we need to employ enhanced sampling as the timescale of ligand unbinding events in this system is prohibitively long: we found in previous studies a mean first passage time of 2.1 s (Dixon et al., 2018), which is six orders of magnitude longer than the reach of conventional MD sampling. In this work, we implement the REVO resampling method, based on weighted ensemble (WE) framework, to encourage the sampling of rare unbinding/rebinding events. WE accelerates the sampling of rare events using an ensemble of trajectories that are each assigned a statistical weight (Huber and Kim, 1996). The ensemble is integrated forward in time in a parallel fashion, and periodically “resampled” by cloning certain trajectories and merging others. When a trajectory is cloned, its weight is divided amongst the clones, but the multiple copies of the trajectory go on to evolve independently. By repeatedly cloning trajectories that are in undersampled regions of space we can obtain statistics on very long-timescale events using only short-timescale simulations.

The REVO resampling method (Resampling Ensembles by Variation Optimization) was designed to efficiently perform cloning and merging operations on small ensembles of trajectories that are evolving in high-dimensional spaces (Donyapour et al., 2019). This is valuable in situations where it is difficult to define one or two progress variables that capture the long-timescale events of interest. In REVO, coupled cloning and merging operations are proposed (e.g., clone trajectory *i*, and merge trajectories *j* and *k*) and are accepted or rejected based on an objective function called the “trajectory variation”:

(3) $$\begin{array}{l} V=\underset{i}{\sum }V_{i}=\underset{i}{\sum }\underset{j}{\sum }{\left(d_{ij}/d_{0}\right)}^{\alpha }\phi_{i}\phi_{j} \end{array}$$

where *d*_*ij*_ is the distance between trajectories *i* and *j*, α and *d*_0_ are parameters, and ϕ_*x*_ is a function that measures the importance, or “novelty” of a trajectory *x*, which in our work here is strictly a function of the weight of the trajectory: ϕ_*i*_ = log *w*_*i*_ − *C*, where *w*_*i*_ is the weight of trajectory *i* and *C* is a constant. Trajectories with the highest *V*_*i*_ values in Equation (3) are chosen for cloning, and those with the lowest *V*_*i*_ are chosen for merging. More information about the algorithm can be found in previous work (Donyapour et al., 2019).

We run separate simulations for the binding and unbinding processes. In our unbinding simulations, the ligands start in the bound state and are terminated as they unbind. In the rebinding simulations, the ligands start in the unbound state and are terminated as they bind. The distance function (*d*_*ij*_) we use in Equation (3) is different for these two simulation types. For the unbinding simulations, we superimpose the hosts from trajectories *i* and *j*, and then compute the root mean squared distance (RMSD) between the guest molecules, without any further alignment (Dickson and Lotz, 2016, 2017),. As there is 4-fold symmetry in this system, we perform the alignment four times (once for each symmetrically-equivalent mapping) and use the smallest such distance as *d*_*ij*_. For the rebinding simulations, we calculate the distance to the native state for each trajectory (*d*_*native*_(***X***_*i*_)), which again takes into account the four symmetry mappings, using the lowest such distance. The distance between trajectories *i* and *j* is then calculated as *d*_*ij*_ = |1/*d*_*native*_(***X***_*i*_) − 1/*d*_*native*_(***X***_*j*_)|, where the inverse is used to prioritize differences between small values of *d*_*native*_.

### 2.4. Calculating Rates by Ensemble Splitting

A major advantage of the REVO method, much like other weighted ensemble methods, is that it can calculate kinetic parameters in real time as the simulation progresses. This is achieved by running separate simulations for the binding and unbinding processes, and in each case, measuring the trajectory flux into the opposite basin (Dickson et al., 2009, 2011; Vanden-Eijnden and Venturoli, 2009; Costaouec et al., 2013; Suárez et al., 2014). The unbound basin is defined as the set of structures where the closest host-guest interatomic distance is >1 nm, following previous work (Dickson and Lotz, 2016, 2017; Lotz and Dickson, 2018). The bound basin is defined as the set of structures where the guest RMSD (compared to the native structure) is <0.1 nm after aligning to the host. Again, this RMSD measurement takes into account the four symmetry-equivalent mappings of OA.

In our studies, the binding and rebinding REVO simulations are conducted separately. However, the methodology of obtaining on and off rates is essentially the same. After each dynamics step, if a walker has entered the opposite basin, as described above, its weight is recorded and its structure is “warped” back to the starting structure at the beginning of the simulation. The atomic coordinates are set to the starting structure and the velocities are reinitialized; however, the weight of the trajectory remains the same. Before the warping event to the starting structure, the structure of the walker is recorded and is referred to as an “exit point.” In our unbinding simulations, the initial starting structure is the initial bound pose provided. In our rebinding simulations, the initial starting structure is chosen from a set of exit points generated from the unbinding simulations. Therefore, the unbinding analyses were performed prior to initialization and the subsequent running of our rebinding simulations.

The off and on rates are calculated by using the flux of trajectories into either the unbound or bound state, respectively.

(4) $$\begin{array}{l} k_{\text{off}}\left(t\right)=\frac{\sum_{i}w_{i}}{t} \end{array}$$

(5) $$\begin{array}{l} k_{\text{on}}\left(t\right)=\frac{\sum_{i}w_{i}}{Ct} \end{array}$$

where the sum is over the set of “warped” trajectories, *t* is the elapsed simulation time, and *C* is the concentration of ligand, computed as 1/*V* where *V* is the box volume. The box volume was approximately 80.2 nm^3^, corresponding to a concentration of ligand of 0.0207 M.

There are a few key differences between the REVO simulations discussed here and our previous studies (Dixon et al., 2018). For both the unbinding and rebinding simulations in this study, the total simulation time is 2.25 times longer compared to our previous study, as our current unbinding and rebinding simulations were run for 4,500 and 450 cycles, respectively. Additionally, ten independent unbinding simulations were run for each of the four friction coefficients, whereas our previous study only ran five independent simulations for each starting pose. However, only five independent rebinding simulations were run for each of the coefficients, as we observe much less variation in the *k*_on_ estimates. Finally, 48 walkers were used in both studies and the time per cycle is consistent, where the unbinding simulations are 20 ps/cycle and the rebinding simulations are 200 ps/cycle.

### 2.5. Calculating Electrostatic Interaction Energies

The electrostatic energy between the host and guest molecules for use in the second correction term was calculated as: $E_{\text{int}}=\frac{1}{4\pi \epsilon_{w}}\frac{Q_{i}Q_{j}}{r_{ij}}$ where *Q*_*a*_ is the partial charge of atom *a* used in the force field during simulation. *r*_*ij*_ is the interatomic distance between atoms *i* and *j* calculated by using the minimum image convention. $\epsilon_{w}=6.88\times 10^{-10}$ F/m is the permittivity of water at 300 K calculated by linear interpolation of the water dielectric constant at 298.15 and 303.15 K (Archer and Wang, 1990).

## 3. Results

### 3.1. Derivation of Correction Terms

The binding free energy can be calculated using the rate constants *k*_on_ and *k*_off_ as $\Delta G=G_{\text{bound}}-G_{\text{unbound}}=-kT\ln\;K_{eq}C_{0}=-kT\ln\;\frac{C_{0}k_{\text{on}}}{k_{\text{off}}}$, where *K*_*eq*_ is the binding equilibrium constant, *C*_0_ is the reference concentration of 1 mol/L, *k* is Boltzmann's constant and *T* is the temperature in Kelvin. While this relationship is correct in the macroscopic limit, it fails to account for the box size and the volume of the unbound state in finite simulation environments with periodic boundary conditions. Here we derive a more accurate expression for the binding free energy that accounts for the finite box size in a typical MD simulation.

Our starting point is an expression for *K*_*eq*_, which is valid for a dilute solution in thermodynamic equilibrium. We use the notation of Woo and Roux (see Equation 4 from Woo and Roux, 2005):

(6) $$\begin{array}{l} K_{eq}=\frac{\int_{\text{bound}}d1\int d\text{X}e^{-\beta U}}{\int_{\text{bulk}}d1\delta \left({\text{r}}_{1}-{\text{r}}_{1}^{*}\right)\int d\text{X}e^{-\beta U}} \end{array}$$

where *U* is the internal energy of the system, β = 1/*kT* is the inverse temperature, **r**_1_ is the center of mass of the ligand (referred to as a “guest” molecule) and ${\text{r}}_{1}^{*}$ is an arbitrary position of the guest in the bulk. Note that *d***1** integrates over the guest positions, and *d***X** integrates over everything else: the host and the solvent degrees of freedom. Note also that *K*_*eq*_ has units of volume, as the delta function constraining the center of mass in the denominator removes three spatial degrees of freedom.

**Figure 2:**
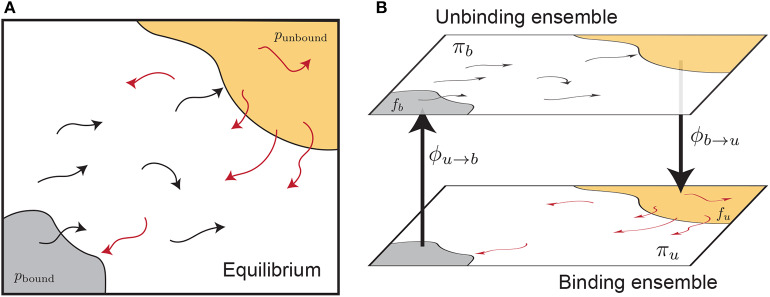
Splitting an equilibrium ensemble **(A)** into two history-dependent ensembles using basins. The bound and unbound basins are shown in gray and light orange, respectively. The unbinding ensemble (**B**, top) contains all trajectories that last visited the bound basin, which are shown in black. The binding ensemble (**B**, bottom, also referred to as the “rebinding” ensemble) contains all trajectories that last visited the unbound basin, shown in red. Simulations in a given ensemble are terminated once they reach the destination basin and thus switch ensembles. The trajectory flux between ensembles is denoted by ϕ_*u*→*b*_ and ϕ_*b*→*u*_. The quantity π_*b*_ refers to the probability of the entire top ensemble, and the quantity *f*_*b*_ denotes the probability of the bound basin within the unbinding ensemble.

Here we examine the calculation of free energies using rates determined from split ensemble calculations (Figure 2, see section 2.4 for more details). We denote the probability of these two ensembles as π_*b*_ and π_*u*_, where π_*b*_ + π_*u*_ = 1, and:

(7) $$\begin{array}{l} \frac{\pi_{b}}{\pi_{u}}=\frac{\phi_{u\to b}}{\phi_{b\to u}} \end{array}$$

where ϕ_*a*→*b*_ is the time-averaged flux from the *a* ensemble to the *b* ensemble (i.e., across the dotted lines in Figure 2). The equilibrium probability of a position **X** can be obtained by combining estimates from both ensembles:

(8) $$\begin{array}{l} p\left(\text{X}\right)=p_{u}\left(\text{X}\right)\pi_{u}+p_{b}\left(\text{X}\right)\pi_{b} \end{array}$$

where *p*_*a*_(**X**) is the probability of conformation **X** in ensemble *a*, which is normalized such that ∫*p*_*a*_(**X**)*d***X** = 1.

Let us define the bound state as the domain of the integral in the numerator of Equation (6), and the unbound state as a set of structures considered unbound in simulation (not the same as the bulk state in Equation 6). These states are shown as shaded regions in Figure 2. The ratio of the probabilities of these two states, at equilibrium, is given by:

(9) $$\begin{array}{l} \frac{p_{\text{bound}}}{p_{\text{unbound}}}=\frac{\int_{\text{bound}}d1\int d\text{X}e^{-\beta U}}{\int_{\text{unbound}}d1\int d\text{X}e^{-\beta U}} \end{array}$$

which can also be calculated in our ensemble splitting simulations:

(10) $$\begin{array}{l} \frac{p_{\text{bound}}}{p_{\text{unbound}}}=\frac{\pi_{b}\int_{\text{bound}}p_{b}\left(\text{X}\right)d\text{X}}{\pi_{u}\int_{\text{unbound}}p_{u}\left(\text{X}\right)d\text{X}}=\frac{\pi_{b}f_{b}}{\pi_{u}f_{u}} \end{array}$$

where *f*_*a*_ is the probability of the basin state within ensemble *a*.

Expanding Equation (6) we have:

(11) $$\begin{array}{l} K_{eq}=\frac{\int_{\text{bound}}d1\int d\text{X}e^{-\beta U}}{\int_{\text{unbound}}d1\int d\text{X}e^{-\beta U}}\frac{\int_{\text{unbound}}d1\int d\text{X}e^{-\beta U}}{\int_{\text{bulk}}d1\delta \left({\text{r}}_{1}-{\text{r}}_{1}^{*}\right)\int d\text{X}e^{-\beta U}}\\ \;=\frac{\pi_{b}f_{b}}{\pi_{u}f_{u}}\frac{\int_{\text{unbound}}d1\int d\text{X}e^{-\beta U}}{\int_{\text{bulk}}d1\delta \left({\text{r}}_{1}-{\text{r}}_{1}^{*}\right)\int d\text{X}e^{-\beta U}}. \end{array}$$

The unbound state in simulation is far enough that the host and guest do not interact directly through van der Waals interactions, although if both molecules carry an explicit charge—as in the example considered here—there could still be significant host-guest electrostatic interactions. To account for these, we introduce another intermediate state with an altered energy function (U^*^) which is the same as *U* except that it does not include electrostatic interactions between the host and the guest:

(12) $$\begin{array}{l} K_{eq}=\frac{\pi_{b}f_{b}}{\pi_{u}f_{u}}\frac{\int_{\text{unbound}}d1\int d\text{X}e^{-\beta U}}{\int_{\text{unbound}}d1\int d\text{X}e^{-\beta U^{*}}}\frac{\int_{\text{unbound}}d1\int d\text{X}e^{-\beta U^{*}}}{\int_{\text{bulk}}d1\delta \left({\text{r}}_{1}-{\text{r}}_{1}^{*}\right)\int d\text{X}e^{-\beta U}} \end{array}$$

(13) $$\begin{array}{l} =\frac{\pi_{b}f_{b}}{\pi_{u}f_{u}}{\langle e^{\beta E_{\text{int}}}\rangle }_{\text{unb}}^{-1}\frac{\int_{\text{unbound}}d1\int d\text{X}e^{-\beta U^{*}}}{\int_{\text{bulk}}d1\delta \left({\text{r}}_{1}-{\text{r}}_{1}^{*}\right)\int d\text{X}e^{-\beta U}} \end{array}$$

where $E_{\text{int}}=U-U^{*}$ and the subscript “unb” indicates an ensemble average over structures in the unbound state obtained with the normal energy function *U*. Note the final step used the relation:

(14) $$\begin{array}{l} \frac{\int_{\text{unbound}}d1\int d\text{X}e^{-\beta U^{*}}}{\int_{\text{unbound}}d1\int d\text{X}e^{-\beta U}}=\frac{\int_{\text{unbound}}d1\int d\text{X}e^{\beta E_{\text{int}}}e^{-\beta U}}{\int_{\text{unbound}}d1\int d\text{X}e^{-\beta U}}={\langle e^{\beta E_{\text{int}}}\rangle }_{\text{unb}}. \end{array}$$

We can now reasonably assume that the guest in the unbound state is non-interacting with the host. This allows us to write *e*^−β*U*^ as $e^{-\beta U_{G}}e^{-\beta U_{HS}}$, where *U*_*G*_ are the terms in the energy function that depend only on the coordinates of the guest, and *U*_*HS*_ are terms that only depend on the host and the solvent. We can then pull the integral $\int d\text{X}e^{-\beta U_{HS}}$ out of the numerator and denominator of the last term of Equation (11):

(15) $$\begin{array}{l} \frac{\int_{\text{unbound}}d1\int d\text{X}e^{-\beta U^{*}}}{\int_{\text{bulk}}d1\delta \left({\text{r}}_{1}-{\text{r}}_{1}^{*}\right)\int d\text{X}e^{-\beta U}}=\frac{\int_{\text{unbound}}d1e^{-\beta U_{G}}}{\int_{\text{bulk}}d1\delta \left({\text{r}}_{1}-{\text{r}}_{1}^{*}\right)e^{-\beta U_{G}}}. \end{array}$$

The bottom integral has the center of mass of the ligand fixed and is only over internal and rotational degrees of freedom of the ligand. This can also be separated and removed from the numerator, which simplifies the ratio to be the volume of the unbound state, defined as:

(16) $$\begin{array}{l} V_{\text{unbound}}=\frac{\int_{\text{unbound}}d1e^{-\beta U_{G}}}{\int_{\text{guest}}d{\text{G}}_{1}e^{-\beta U_{G}}}=\int_{\text{box}}dR\phi_{u}\left(R\right) \end{array}$$

where we use **G**_1_ to denote the internal and rotational degrees of freedom of the guest that remain after specification of **r**_1_. The quantity ϕ_*u*_(**R**) is the fraction of conformers with center of mass **R** that satisfy the unbound boundary conditions: here, that the guest atoms are all farther than a cutoff distance of 1 nm away from the host. This integral can be calculated by Monte Carlo, where a center of mass position and orientation of the ligand is randomly generated, and the number of successful unbound conformers is recorded:

(17) $$\begin{array}{l} V_{\text{unbound}}=V_{\text{box}}\frac{N_{\text{unbound}}}{N_{\text{trials}}}. \end{array}$$

Note that for large boxes *V*_unbound_ ≈ *V*_box_.

Putting this all together we have:

(18) $$\begin{array}{l} K_{eq}=\frac{\pi_{b}f_{b}}{\pi_{u}f_{u}}{\langle e^{\beta E_{\text{int}}}\rangle }_{\text{unb}}^{-1}V_{\text{unbound}}, \end{array}$$

which differs from the straightforward interpretation used in our previous work (Dixon et al., 2018):

(19) $$\begin{array}{l} K_{eq}^{0}=\frac{\pi_{b}}{\pi_{u}\left[L\right]}=\frac{\pi_{b}}{\pi_{u}}V_{\text{box}} \end{array}$$

Using Δ*G* = −*kT*ln(*K*_*eq*_*C*_0_), we have:

(20) $$\begin{array}{l} \Delta G=\Delta G^{0}-kT\ln\;\left(\frac{f_{b}}{f_{u}}\right)+kT\ln{\langle e^{\beta E_{\text{int}}}\rangle }_{\text{unb}}-kT\ln\;\left(\frac{V_{\text{unbound}}}{V_{\text{box}}}\right) \end{array}$$

which explicitly shows Δ*G* as the sum of Δ*G*^0^ $=-kT\ln\left(K_{eq}^{0}C_{0}\right)$ and the three newly derived correction terms. The first term will go to zero in the limit that the basin states are chosen to represent the vast majority of the probability in both the binding and unbinding ensembles. In other words, this term goes to zero when both *f*_*b*_ and *f*_*u*_ approach one. The second term is likely to only be non-negligible in the case of explicitly charged host and guest molecules and regardless would go to zero as the definition of the unbound state is moved to farther and farther distances. The third term would also go to zero for large simulation boxes, but in practice this is often not feasible due to computational constraints. Consequently, *V*_unbound_/*V*_box_ could be much less than one, introducing a correction in the positive direction. Below we calculate these three correction terms and apply them to free energy calculations.

### 3.2. Extended Trajectory Ensembles With Lower Friction Coefficients

In previous work, we used a Langevin integrator with a value of γ = 1 ps^−1^ for the friction coefficient. As the simulations already have explicit solvent, this adds extra friction into the system that is not physical. Here we investigate whether reducing γ to values less than one will significantly affect our rate calculations. We thus run a set of trajectory ensembles at multiple values of γ and extend each ensemble to be larger and longer than those published in our prior study (Dixon et al., 2018) to more fully examine questions of convergence.

**Figure 3:**
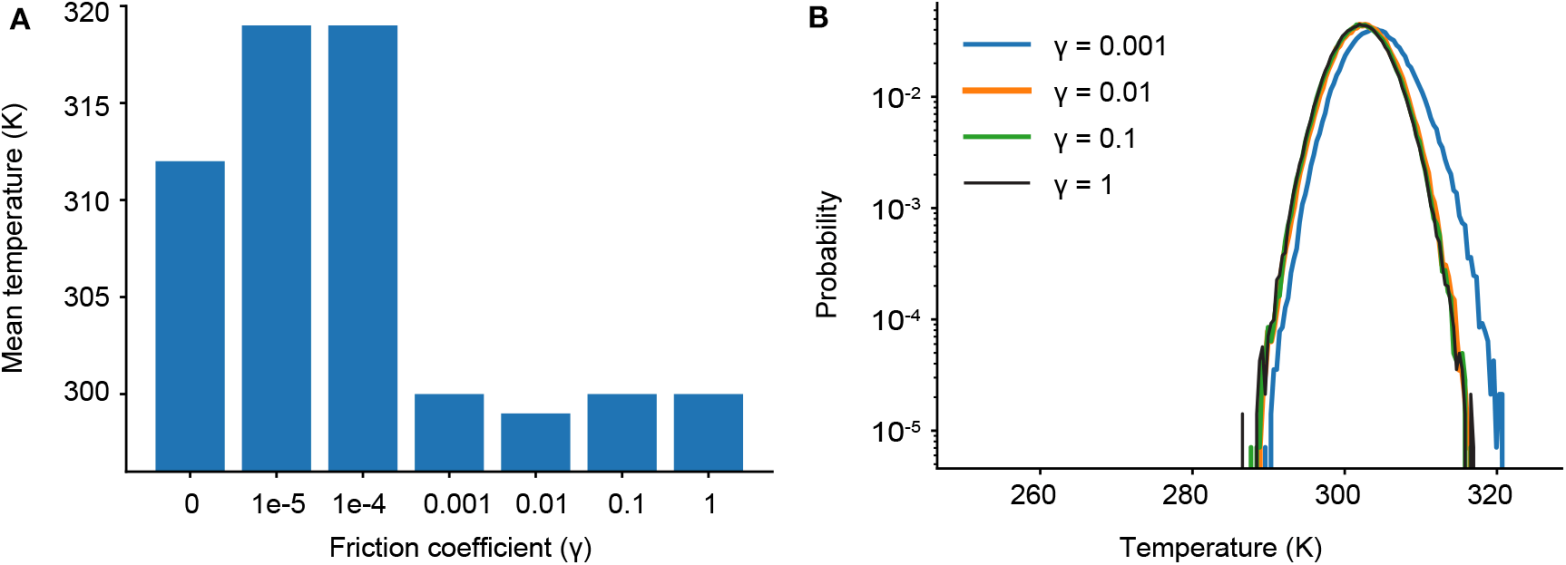
**(A)** Average temperatures observed in short simulations for different friction coefficients (γ). **(B)** Probability distributions of observed temperatures from ensembles of longer simulations with different γ.

As γ governs the coupling to the Langevin thermostat, we determine the minimum value of γ where our target temperature (300 K) is maintained. We first ran a series of short simulations (one 10 ns trajectory for each γ) and find that temperature control is completely lost for friction coefficients less than γ = 0.001 (Figure 3A). We then ran longer simulations for γ = 1, 0.1, 0.01, and 0.001, examining not only the mean temperature, but the probability of significant temperature fluctuations, which could spur anomalous results in our ligand dissociation simulations. Figure 3B shows the probability distribution of observed temperatures over an ensemble of 240 trajectories run for 90 ns each. For γ = 0.01, 0.1 and 1 ps^−1^, the temperature distribution is normally distributed around the mean (300 K) as seen by the parabolic curves on a log scale. Temperature control is not fully maintained for γ = 0.001 ps^−1^, as shown by a rightward shift and slight widening of the parabolic distribution. We thus restrict our analysis to three values of the friction coefficient: γ = 0.01, 0.1, and 1 ps^−1^.

**Figure 4:**
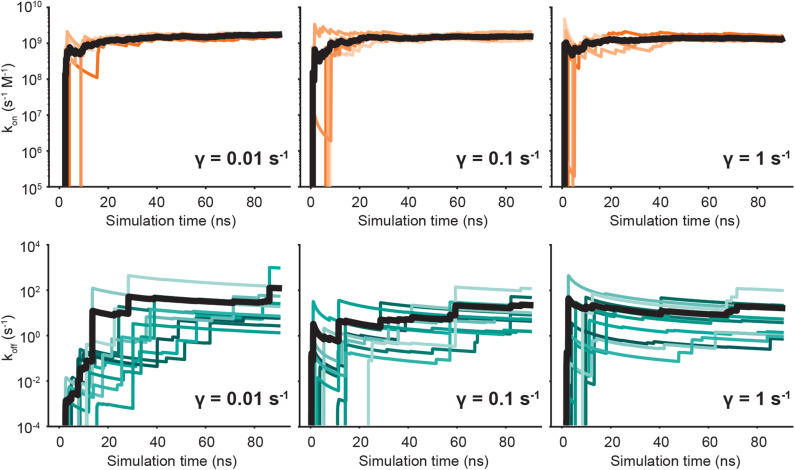
Predicted on- **(top)** and off-rates **(bottom)** as a function of simulation time. Each panel is labeled according to the friction coefficient used for that set of simulations. The independent simulations are shown in shades of orange (*k*_on_) and blue (*k*_off_), and the averages are depicted by bold black lines.

We run both unbinding and rebinding REVO simulations for the OAG6 system. For unbinding, we ran 10 simulations for each of the three friction coefficients; for rebinding, we ran five simulations for each coefficient, yielding a total of 30 simulations for unbinding and 15 simulations for rebinding. A set of binding and unbinding simulations were also run for γ = 0.001—despite the impaired temperature control—which are reported in the Supplemental Information. The estimates for the unbinding and binding fluxes are depicted in Figure 4, where each curve represents an individual REVO simulation. The averages, illustrated with a bolded line, are calculated by averaging the trajectory flux over the entire set of simulations for that value of γ. The upward jumps on these plots indicate that an exit point was recorded that has a higher weight than was previously observed.

By reducing γ to values <1, we observed no change in the binding rates, and small changes to the unbinding rates which are on the border of significance. With regard to unbinding rates, the two largest friction coefficients yielded the smallest error and similar *k*_off_ values, where γ = 1 yielded an average off rate of 16.4 s^−1^ and γ = 0.1 yielded an off rate of 11.5 s^−1^. The off-rate increased by 10-fold for γ = 0.01, although this is mostly driven by exit points observed in a single simulation. In our previous OA-G6 results using γ = 1, we calculated an unbinding rate of 0.48 s^−1^ which slightly differs from the value calculated in this study using γ = 1 (Table 1). Unbinding rates for γ = 0.001 ps^−1^ were approximately 1000-fold higher, although these are known to be affected by a higher average temperature (Supplemental Information). Taking a closer look at the binding rates, we saw no discernible difference across the friction coefficients. The binding rate was approximately 10^9^ s^−1^ M^−1^, for all friction coefficients, which was about 5-fold larger when compared to our previous study using γ = 1. For both binding and unbinding rates we have more confidence in the results obtained here, as they are based on more extensive simulation data.

**Table 1:** Binding and unbinding rates as a function of friction coefficient (γ).

	***k*_on_** (**10^8^*M*^−1^*s*^−1^)**	***k*_off_ (*s*^−1^)**
γ = 0.01	17 ± 1	122 ± 94
γ = 0.1	16 ± 2	22 ± 12
γ = 1	13 ± 1	16.4 ± 9.4
Dixon et al. (2018) (γ = 1)	2.8 ± 1.0	0.48 ± 0.11

*The uncertainties shown use the standard error of the mean calculated from 5 and 10 independent REVO runs for binding and unbinding, respectively. The quantities from Dixon et al. (2018) were obtained with 5 REVO runs that used different initial conformations, each of which were 2,000 cycles in length*.

For both the unbinding and rebinding simulations, across all friction coefficients, we observed at least 1,000 warping events (Figure S4). As expected, we observe that rebinding occurs with a much higher probability when compared to unbinding, by several orders of magnitude. The unbinding walker weights are limited at the low end by the minimum walker probability (*p*_*min*_), which is set to 10^−12^. The rebinding walker weights are limited at the high end by the maximum walker probability (*p*_*max*_), which is set to 10^−1^. respectively. Figure S4 shows that the 10-fold larger unbinding rate fro γ = 0.01 was largely due to a single unbinding point in a single simulation, which underscores the sensitivity and uncertainty of rate calculations using trajectory fluxes. Figure S2 shows unbinding fluxes for γ = 0.001, which is known to have elevated temperatures. There we see a large number of high-weight unbinding events in two different simulations, leading to the 1,000-fold increase in *k*_off_.

### 3.3. Free Energy Estimates, Correction Terms, and Comparison With Previous Benchmarks

**Figure 5:**
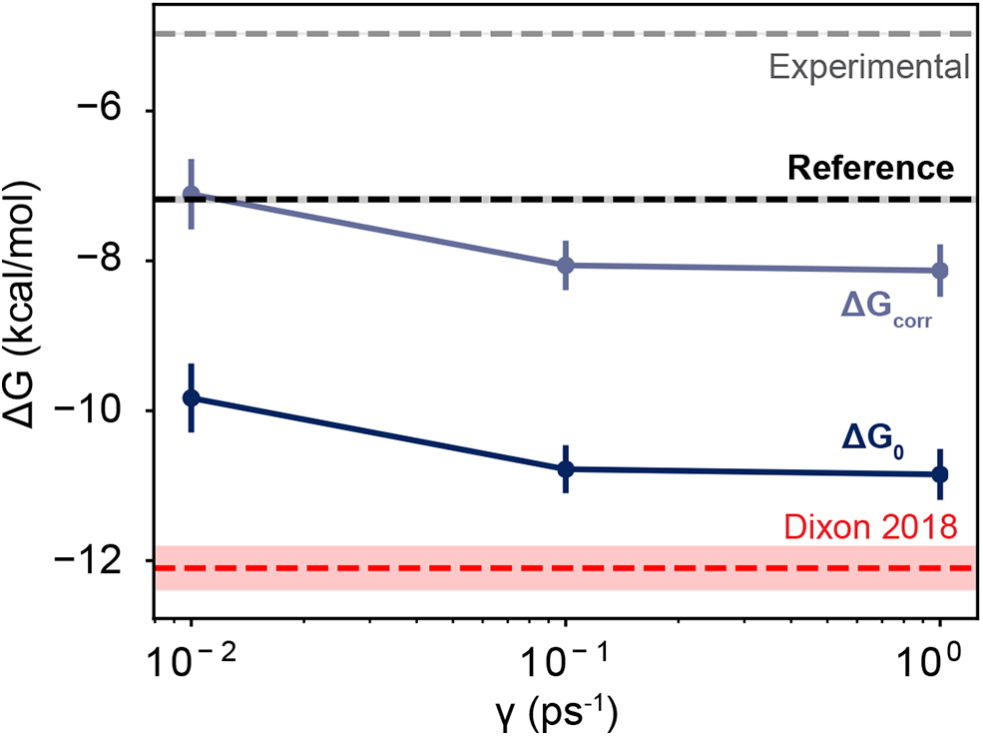
Free energies as a function of friction coefficient. The dark blue line shows the uncorrected free energies calculated at three different γ values. The light blue line shows the corrected values, which are shifted upwards by 2.72 kcal/mol. The thin red line shows the value reported in Dixon et al. (2018), which employed a friction coefficient of 1.0 ps^−1^ and used a smaller dataset than is reported here. The black horizontal line shows the value of a computational reference computed using alchemical perturbation, reported in Rizzi et al. (2020). The dashed gray line shows the experimental measurement, reported in Sullivan et al. (2019). The shaded area for each line shows its associated uncertainty, which is less than the line thickness for the computational reference and the experimental measurement.

As the friction coefficient unevenly affected the rates of binding and unbinding, there was a net effect on the binding free energies. As shown in Figure 5 and Table 2, the binding free energy increases as the friction coefficient is lowered, independent of the free energy correction terms derived in section 3.1. Table 2 shows the free energies computed using the averaged fluxes across all simulations at each γ value. For all friction coefficients, the calculated free energy was always higher than that from our previous study (−12.1 kcal/mol; red line), even for γ = 1, signifying that extending the simulation time aided in predicting experimentally determined binding free energies.

**Table 2:** Raw (Δ*G*^0^) and corrected (Δ*G*_corr_) free energy values using simulation data from three different friction coefficients.

	**Δ*G*^0^ (kcal/mol)**	**Δ*G*_corr_ (kcal/mol)**
γ = 0.01	−9.83 ± 0.46	−7.11 ± 0.47
γ = 0.1	−10.78 ± 0.32	−8.06 ± 0.33
γ = 1	−10.85 ± 0.34	−8.13 ± 0.36
Dixon et al. (2018) (γ = 1)	−12.1 ± 1.0	−9.38 ± 1.0
Comp. (Rizzi et al., 2020)	–	−7.0 ± 0.1
Exp. (Sullivan et al., 2019)	–	−4.97 ± 0.02

*Values are in kcal/mol and uncertainties are calculated using propagation of the standard error of the mean*.

The correction terms are calculated using data obtained from the simulations, but they are mostly functions of geometric properties of the simulation box and boundary conditions, and are not expected to change as a function of γ. The first term, −*kT* ln *f*_*b*_/*f*_*u*_, was calculated to be 0.74 ± 0.10 kcal/mol, with *f*_*b*_ and *f*_*u*_ taking on values of 0.157 and 0.54, respectively. As described in section 3.1, *f*_*b*_ is the probability of the being in the bound basin given that you are in the unbinding ensemble, which is calculated using the sum of the weights of trajectories in the bound basin, divided by the total sum of the weights of the trajectories considered. The *f*_*b*_ value in particular was lower than expected, indicating that our definition of the bound state might be too restrictive, even though we did account for all symmetry-equivalent conformations in our calculation of *f*_*b*_.

The second term, $+kT\ln{\langle e^{\beta E_{\text{int}}}\rangle }_{\text{unb}}$, was calculated to be 1.64 ± 0.002 kcal/mol. This was calculated by determining the electrostatic interaction energies (see section 2.5) for the set of unbound states observed in the rebinding simulations. The expectation value in the correction term again accounted for trajectory weights and was computed using 71,428 interaction energy measurements that were selected from the unbound ensemble. The uncertainty was computed as the standard error of the mean of this set of energies. To calculate the third correction term, $-kT\ln\;\left(\frac{V_{\text{unbound}}}{V_{\text{box}}}\right)$, we directly estimated *V*_unbound_/*V*_box_ using the Monte Carlo procedure described in section 3.1. The ratio was computed as 0.56 ± 0.0037 using five batches of 10,000 trials each, where the uncertainty is the standard error of the mean across the sets of trials.

Together these three terms sum to 2.72 kcal/mol, which is a significant correction to the binding free energies computed here. Over half of this comes from the residual electrostatic interaction energy between the host and the guest. Note that both the host and the guest have negative charges, and the residual interaction between the two molecules is repulsive. Turning this interaction off *releases* 1.64 kcal/mol of energy, which lowers the free energy gap between the bound and unbound states. The corrected and uncorrected free energies are shown as a function of γ in Figure 5. For γ ≥ 0.01 the calculated free energies are almost equal to within standard error and the correction terms significantly reduce the error with respect to the computational reference value (Rizzi et al., 2018, 2020).

## 4. Discussion and Conclusion

In this study, we sought to better connect the calculation of binding and unbinding rates with the calculation of binding free energies. The rate calculations measured the microscopic fluxes of trajectories from one basin to another. These fluxes can be visualized in an extended history-dependent conformation space, where trajectories change their “color" based on which basin (“bound” or “unbound”) they have most recently visited (Dickson et al., 2009, 2011; Vanden-Eijnden and Venturoli, 2009; Costaouec et al., 2013; Suárez et al., 2014). The ratio of these rates gives a ratio of two populations: the trajectories that have most recently visited the “bound” basin and the trajectories that have most recently visited the “unbound” basin. The first correction term adjusts this ratio to instead only account for the probability contained within the basins themselves and is particular to rates that are calculated using this history-dependent formalism. The third term can be seen as a volume correction term, which is used to accurately account for the volume in the unbound state. This is done in other approaches where restraints are used, such as methods based on calculation of the potential of mean force (Deng and Roux, 2009). In our case the unbound state cannot be easily approximated by a geometric object, such as the volume of a spherical shell.

The second term accounts for residual interactions in the unbound ensemble. This could be used by other approaches that directly determine free energy differences between bound and unbound conformations, such as Markov state modeling, metadynamics, milestoning, and umbrella sampling. The conventional approach is to define a simulation box that is large enough such that the interactions between the host and guest are negligible in the unbound state. However, this can significantly increase the cost of the simulation. It is worth noting that umbrella sampling results for this system (OA-G6) obtained by Song et al. (2018), −8.50 kcal/mol, were also below both the computational benchmark and the experimental value. Their unbound state was defined as a 20 Å distance between an atom in the guest and a dummy atom in the center of the host, which is roughly comparable to our unbound basin of 10 Å of clearance between the host and the guest. Assuming a similar value for the electrostatic correction term, it would have brought their prediction to −6.86 kcal/mol, which is in line with the computational benchmarks (Rizzi et al., 2020).

The electrostatic term can also be viewed as a sort of “decoupling” between the host and the guest, and it is warranted to discuss similarities and differences with similar procedures in alchemical free energy methods. They are similar in that we are computing a free energy between two Hamiltonians, one in which an interaction is turned off. We could thus use similar techniques for computing these free energy differences, such as thermodynamic integration (Kirkwood, 1935; Bhati et al., 2019), BAR (Gutiérrez et al., 2019), MBAR (Shirts and Chodera, 2008; Bhati et al., 2019), or MM/PBSA (Rifai et al., 2019), although here we effectively use a simple free energy perturbation (FEP) expression (Zwanzig, 1954; Jorgensen and Thomas, 2008). The approaches are different in that we are only considering ensembles of structures where the interactions being turned off are relatively weak. We are assuming here—as is always the case with FEP—that the conformational ensembles of both the host and the guest are highly overlapping between the two Hamiltonians, which considerably simplifies the problem. We also note that although we employ electrostatic decoupling to compute free energies, our simulations still reveal important information about the (un)binding kinetics and mechanism.

Given these correction terms for the binding affinity, it is reasonable to ask if and how the rate constants should be modified. The correction terms each have the effect of “loosening” the interaction, indicating that either the corrected off-rate should increase, or the corrected on-rate should decrease. It is reasonable to assume that lowering the on-rate should account for the vast majority of this correction, as the on-rate measured starts from an unbound conformation that is much closer (a clearance of 1 nm between the host and guest) than is likely in experimental conditions. More accurate calculations of the binding rate can be achieved with better sampling of the unbound state, for instance using the Northrup-Allison-McCammon method (Northrup et al., 1984). It would be interesting to see whether such calculations can recapitulate part of the free energy differences observed here.

We also examined the role that the Langevin integrator plays in the prediction of kinetic and thermodynamic quantities. In particular, we adjusted the friction coefficient (γ), defined in the Langevin integrator, while maintaining the stability of temperature at 300 K. We did not expect that altering the friction coefficient would have an impact on the calculation of equilibrium quantities. As γ does not appear in the Hamiltonian of the system, it should not affect the probability of a given microstate *P*(**X**), which is given by the Canonical probability density exp(−β*U*(**X**)). While we did expect it to affect rates, we expected that these effects would offset: that if unbinding was accelerated 10-fold, we would observe the binding process to be sped up by the same factor. However, we observe that the on-rate was very stable as a function of γ, while the off-rate changed slightly. One explanation is that unbinding is a much more rare event when compared to rebinding, and estimates of *k*_off_ were not converged. Lower friction coefficients could be accelerating sampling of these events and making it easier to observe higher probability walkers unbind in our simulations.

Convergence is of utmost priority in weighted ensemble simulations that calculate kinetic quantities. In our previous study, we hypothesized that it was possible that extending the time of the unbinding simulations could capture more high weight walkers exiting from the bound state. Indeed, we observe a higher unbinding flux in this study across all friction coefficients. In Figure 4, we observe large upward jumps, for all γ values, even after 40 ns of simulation time per walker, which was sampling limit in our previous study. These upward jumps, as previously described, signify that an exit point was recorded that has a higher weight than previously observed. This highlights the challenges involved in accurate determination of rate fluxes for rare events. It is worth noting that by using our correction terms to account for small unbound volumes and persistent but small electrostatic interactions in the unbound state, we can keep box sizes small, allowing for better convergence of rate fluxes at fixed computational cost.

Of course the binding free energy alone is still an important quantity for drug design (Homeyer et al., 2014). If one is only interested in the absolute binding free energy, calculating it through the ratio of rates is needlessly complicated; free energy is a state function and thus only depends on the endpoints of the binding pathway. The prediction of *k*_off_ and *k*_on_ themselves is challenging, since they are not state functions: they depend on the transition path ensemble between the bound and unbound state. Sampling of these physical pathways is a large challenge for molecular dynamics, largely due to the long timescales of the binding and release processes. Ensuring that the ratio of rates is consistent with binding free energy calculations—as done here—provides an additional, powerful consistency check. In particular, comparing to well-converged computational benchmarks is more useful than experimental quantities, as we avoid an additional layer of uncertainty associated with the force field used to describe the system.

## Data Availability Statement

The datasets generated for this study are available on request to the corresponding author.

## Author Contributions

AD designed the project. RH, TD, and AD conducted the research, analysis, and wrote the manuscript.

## Conflict of Interest

The authors declare that the research was conducted in the absence of any commercial or financial relationships that could be construed as a potential conflict of interest.
