# Supplementary material for: On Calculating Free Energy Differences Using Ensembles of Transition Paths
Source: Front Mol Biosci. 2020 Jun 5;7:106. doi: 10.3389/fmolb.2020.00106 (PMC7291376; doi:10.3389/fmolb.2020.00106)
Supplement: Supplementary file 1 [file Data_Sheet_1.PDF]

Correction terms for calculating binding free energy using rates from nonequilibrium simulations
Supplemental Information

Robert Hall
Department of Biochemistry & Molecular Biology,
Michigan State University, East Lansing, MI 48824

Tom Dixon and Alex Dickson*
Department of Biochemistry & Molecular Biology,
Michigan State University, East Lansing, MI 48824 and
Department of Computational Mathematics, Science and Engineering,
Michigan State University, East Lansing, MI 48824
(Dated: February 21, 2020)

S1. RATES AND FREE ENERGIES FOR $\gamma = 0.001$

Although the simulations with $\gamma = 0.001$ were found to suffer from impaired temperature control, for completeness we report the unbinding and rebinding simulation results here. Figure S1 shows the fluxes from the binding and unbinding ensembles observed over 5 and 10 simulations, respectively. Although the binding rates were comparable to those observed at higher friction coefficients, the unbinding rates were significantly higher. As shown in Figure S2, these high unbinding weights were observed in only two simulations in our set of 10. The unbinding rate constant calculated under these conditions is $k_{\text{off}} = 18700 \pm 18500$ s^{-1}, which is higher than that calculated at $\gamma = 0.01$ by a factor of ~ 150. This leads to a binding free energy of $\Delta G = -6.77 \pm 0.59$ kcal/mol (before corrections) and a corrected binding free energy of $\Delta G = -4.05 \pm 0.60$ kcal/mol. An extended free energy plot versus γ is shown in Figure S3.

*Electronic address: `alexrd@msu.edu`

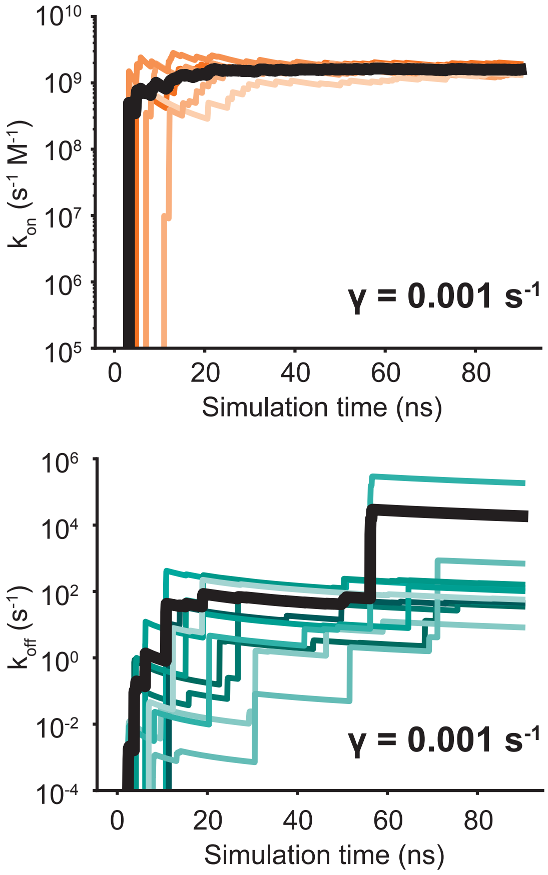

FIG. S1: Binding (top) and unbinding (bottom) fluxes for $\gamma = 0.001$ ps^{-1}. Fluxes are shown for each simulation individually. Parameters are the same as those used for higher γ values in the main text. Average fluxes over the simulations are shown as thick black lines.

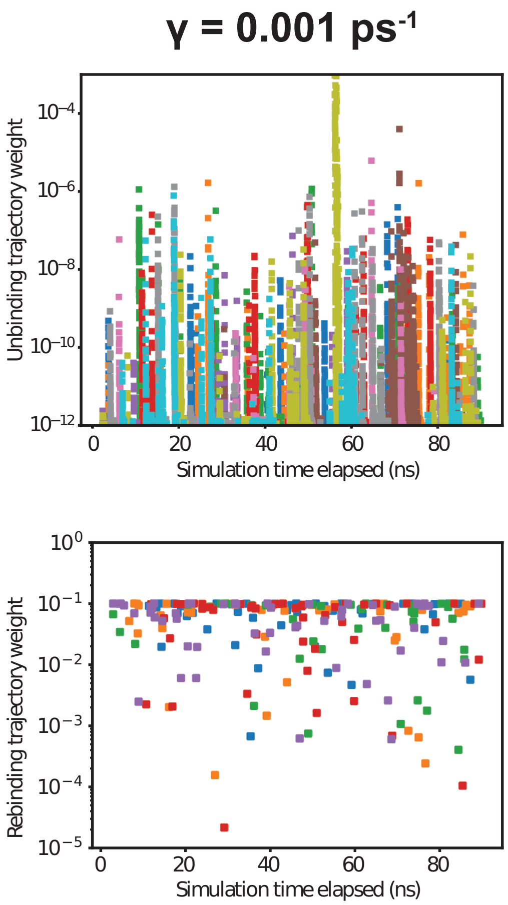

FIG. S2: Weights of warped walkers in unbinding (top) and binding (bottom) REVO simulations for $\gamma = 0.001$ ps^{-1}. Each simulation is shown in a different color. Parameters are the same as those used for higher γ values in the main text.

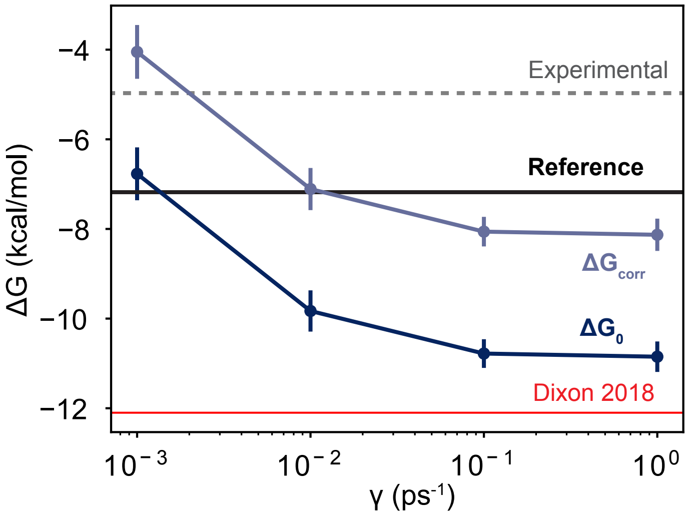

FIG. S3: Free energies as a function of friction coefficient for $\gamma = 0.001$, 0.01, 0.1 and 1.0 ps^{-1}. The dark blue line shows the uncorrected free energies calculated at three different γ values. The light blue line shows the corrected values, which are shifted upwards by 2.72 kcal/mol. The thin red line shows the value reported in Ref. [S1], which employed a friction coefficient of 1.0 ps^{-1} and used a smaller dataset than is reported here. The black horizontal line shows the value of a computational reference computed using alchemical perturbation, reported in Ref. [S2]. The dashed grey line shows the experimental measurement, reported in Ref. [S3].

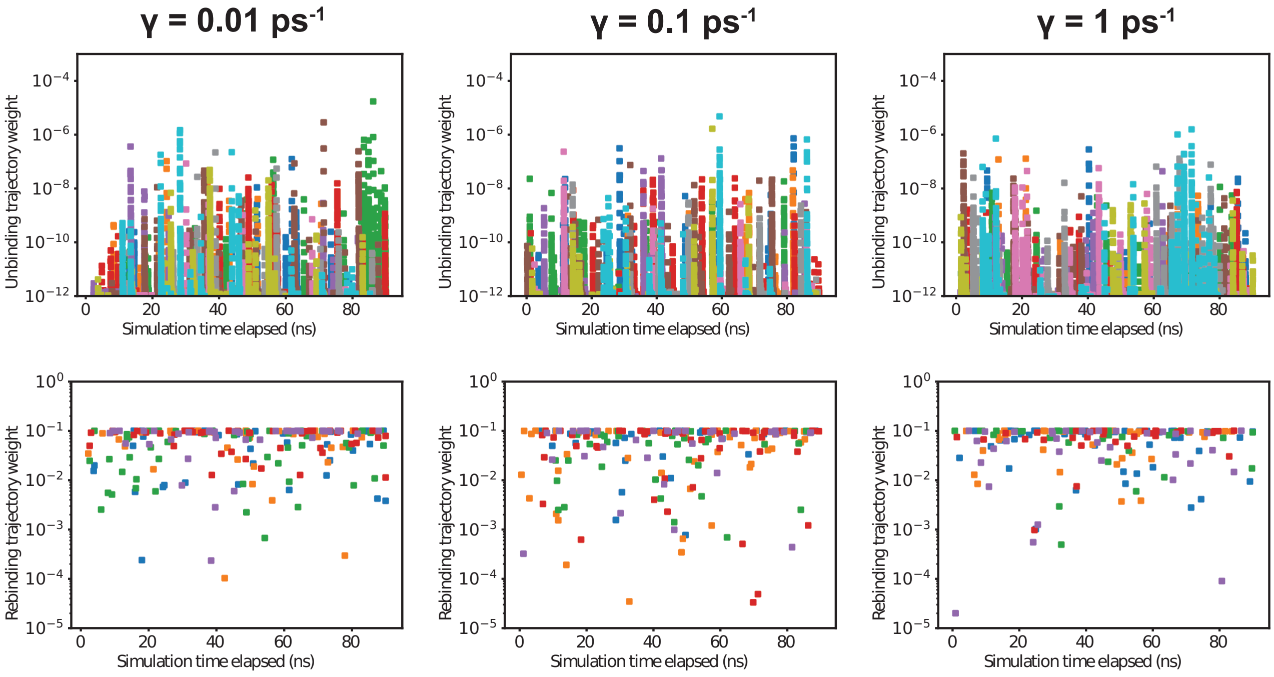

FIG. S4: Weights of warped walkers in unbinding (top) and binding (bottom) REVO simulations for $\gamma = 0.01$, 0.1 and 1.0 ps^{-1}. Each simulation is shown in a different color.

S2. SUPPORTING REFERENCES

[S1] T. Dixon, S. D. Lotz, and A. Dickson, Journal of Computer-Aided Molecular Design **32**, 1001 (2018), ISSN 15734951.
[S2] A. Rizzi, T. Jensen, D. R. Slochower, M. Aldeghi, V. Gapsys, D. Ntekoumes, S. Bosisio, M. Papadourakis, N. M. Henriksen, L. D. Groot, et al., Journal of Computer-Aided Molecular Design pp. 1–33 (2020).
[S3] M. R. Sullivan, W. Yao, and B. C. Gibb, Supramolecular Chemistry **31**, 184 (2019), ISSN 10290478, URL `https://doi.org/10.1080/10610278.2018.1549327`.
